# Weak Power Frequency Magnetic Field Acting Similarly to EGF Stimulation, Induces Acute Activations of the EGFR Sensitive Actin Cytoskeleton Motility in Human Amniotic Cells

**DOI:** 10.1371/journal.pone.0087626

**Published:** 2014-02-05

**Authors:** Xia Wu, Mei-Ping Cao, Yun-Yun Shen, Ke-Ping Chu, Wu-Bin Tao, Wei-Tao Song, Li-Ping Liu, Xiang-Hui Wang, Yu-Fang Zheng, Shu-De Chen, Qun-Li Zeng, Ruo-Hong Xia

**Affiliations:** 1 Physics Department, East China Normal University, Shanghai, China; 2 Bioelectromagnetics Laboratory, Zhejiang University, Hangzhou, China; 3 School of Life Sciences, Fudan University, Shanghai, China; 4 State Key Laboratory of Precision Spectroscopy, East China Normal University, Shanghai, China; National Research Council, Italy

## Abstract

In this article, we have examined the motility-related effects of weak power frequency magnetic fields (MFs) on the epidermal growth factor receptor (EGFR)-sensitive motility mechanism, including the F-actin cytoskeleton, growth of invasive protrusions and the levels of signal molecules in human amniotic epithelial (FL) cells. Without extracellular EGF stimulation, the field stimulated a large growth of new protrusions, especially filopodia and lamellipodia, an increased population of vinculin-associated focal adhesions. And, an obvious reduction of stress fiber content in cell centers was found, corresponding to larger cell surface areas and decreased efficiency of actin assembly of FL cells in vitro, which was associated with a decrease in overall F-actin content and special distributions. These effects were also associated with changes in protein content or distribution patterns of the EGFR downstream motility-related signaling molecules. All of these effects are similar to those following epidermal growth factor (EGF) stimulation of the cells and are time dependent. These results suggest that power frequency MF exposure acutely affects the migration/motility-related actin cytoskeleton reorganization that is regulated by the EGFR-cytoskeleton signaling pathway. Therefore, upon the MF exposure, cells are likely altered to be ready to transfer into a state of migration in response to the stimuli.

## Introduction

Migration is an important property of both normal and tumor cells and relies on the actin cytoskeleton shifting from one state to another. One of the key events as a cell begins migration or metastasis is that its actin cytoskeleton becomes dynamic by developing more-invasive protrusions. Actin assembly drives the extension of protrusion organelles, such as lamellipodia and filopodia, at the leading edge of the cell, accompanied by the dissociation of stress fibers in the cell center. In normal cells, cell motility is involved in many important physiological processes, such as nutrition, chemotaxis, and wound healing [1]–[2]. For a tumor cell, in extreme cases, the active actin cytoskeleton plays a key role not only in migration during metastasis but also in protection from immune surveillance in the stroma surrounding new sites [3]–[4]. One of the key aims of this study is to understand if and how a cell becomes mobile and aggressive in a cytoskeleton-dependent manner in response to environmental stimuli.

Cells exhibit invasive properties that are directly linked to the cellular actin cytoskeleton organization, which is also regulated by epidermal growth factor receptor (EGFR)-related signal pathways. Furthermore, the activation of signaling pathways is essential for triggering the cellular motility mechanism for survival, which is inseparably associated with actin cytoskeleton reorganization. This process is highly orchestrated and involves many actin assembly-regulating proteins (AARPs), including signal proteins, such as fascin, Arp2/3, myosin light chain (MLC), and vinculin etc. These molecules are the downstream signaling proteins in the signaling pathways that regulate the invasive or structural actin cytoskeleton. Among these proteins, fascin, which binds to the filaments in filopodia, plays a key role in establishing these filaments, whose over-expression generally induces greater filopodial growth [5]–[8]. Arp2/3, which is usually found in lamellipodia, acts as a nucleation core for the assembly of new branch filaments, through which the complex stimulates filament polymerization in the cell leading edge [4], [9]. Furthermore, MLC, a myosin regulatory protein that binds to myosin II [10], mediates a variety of events, including the formation of stress fibers [10]–[11], changes in cell shape [12], and cell contraction [12]–[13], by integrating with the F-actin in stress fibers [13]. MLC content that is inseparable from F-actin is consistent with the contractility of stress fibers [1], and vinculin plays an important role in focal adhesions [4] during cell spreading.

EGFR is a cytoskeleton-binding protein. The F-actin microfilaments of the cytoskeleton bind to EGFRs at sites where AA_984–990_ overlaps Tyr_992_, which are important for initiating downstream signaling upon EGFR activation. Actin polymerization is, in turn, regulated by initiating EGFR binding to the cytoskeleton [14]–[15]. Actin filaments act as a scaffold to which the EGF-induced signaling complex binds [16].Morphological changes and actin cytoskeleton reorganization are some of the earliest responses to EGFR activation [17]. Actin-based structures and their functions are intimately associated with their dynamic properties and depend on the spatial distribution and activities of AARPs. A dynamic cytoskeleton is a feature of migrating cells. It was widely found that cells in healing wounds [1]–[2] migrate at a high speed to accelerate wound closure, while tumor cells, especially those undergoing tumorigenesis [3], are also often highly mobile in vivo, which is a major problem in tumor therapy.

The mechanisms of cell migration are affected by numerous physical and chemical external factors, including electromagnetic fields (EMFs). EMFs have been applied in medical therapies and were reported to be able to improve wound healing and tissue repair [18]–[19] and to accelerate the proliferation of osteoblasts to promote fracture healing [19]–[20].

However, there has been concerned that intensive exposure to EMFs is hazardous to people in professional work groups. An alert was raised over exposure to relatively low-intensity magnetic fields when a report was released indicating that long-term exposure to weak public power frequency magnetic fields (MFs) may cause health problems [21]. The question of whether there is any consequence of exposure to weak power frequency MFs for a healthy human has become a current environmental health issue, resulting in massive amounts of evidence and opinions from studies that have focused on the effects of power frequency MF on cell biology. Among these, several studies revealed that weak power frequency MFs initiate EGFR signaling, indicating that membrane receptor EGFR could be a candidate to bridge the power frequency MF signal to the cell. It has been found that power frequency MF induces cell-surface EGFR and isolated EGFR clusterings in the absence of ligand binding in different cell lines [22]–[23]. It has also been demonstrated by our previous work that EGFR clustering induced by a weak power frequency MF was blocked by the EGFR tyrosine kinase (TK) inhibitor PD153035 (PD), inducing cytoskeletal changes in different cell lines [22]–[23] suggesting that power frequency MF activates upstream EGFR signaling pathways [24].

It’s known that modifications in cell shape and morphology relates to different actin distribution patterns [25] and that 50 Hz MF induced alternations in adhesion pattern in HaCaT cells in recent year, observed by multiple techniques such as SEM [25], confocal, and AFM [26]. Meanwhile, we have reported or observed the effects of 30-min, 0.1–0.5 mT power frequency MF exposures on morphological changes of the cytoskeleton in FL and CHL cell lines [22]–[23], and found that the MF as low as 0.2 mT induced overt cytoskeletal changes (data not shown in this article). Considering that the IRPA safety limit for power frequency MF is 0.2 mT for the public and 1 mT for professionals [27], our previous results suggests that a weak power frequency MF may interfere the cytoskeleton-motility mechanism. In this article, our work focuses on the morphological and molecular effects of 0.4-mT power frequency MF exposure on the invasive structure-related actin cytoskeleton reorganization associated with EGFR activation in an FL cell line. The aim is to understand whether MF exposure plays a critical role, similar to that of EGF, in evoking an EGFR-dependent cytoskeletal invasive structure transition through the MF-driven EGFR activation.

## Results

### 1. Power Frequency MF Induces the Growth of New Invasive Structures and Affects the Spatial Distribution of F-actin in vivo

**Figure 1:**
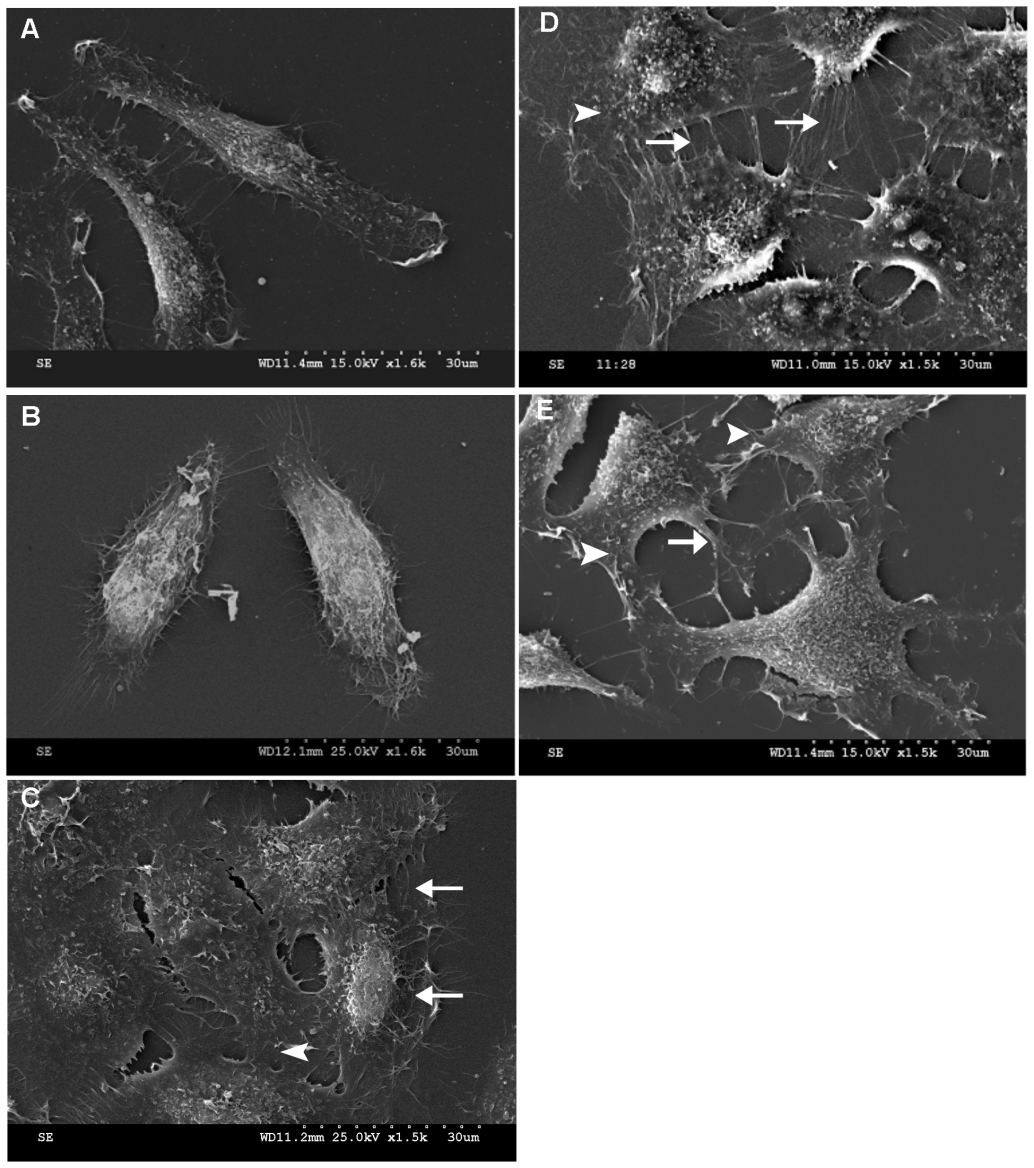
MF induces increases in filopodia-like protrusions and focal adhesions inFL cells. A: negative control (N-con); B: sham-exposed (Sham); C: treated with100 nMEGF (EGF); D: exposed to 0.4 mT MF for 30 min (MF); E: pre-treated with PD, then exposed to MF (+PD+ MF). Arrow: appearance of filopodia; arrowhead: lamellipodia. The detailed information of experimental conditions and repeating numbers of samples is seen in Table 1.

**Figure 2:**
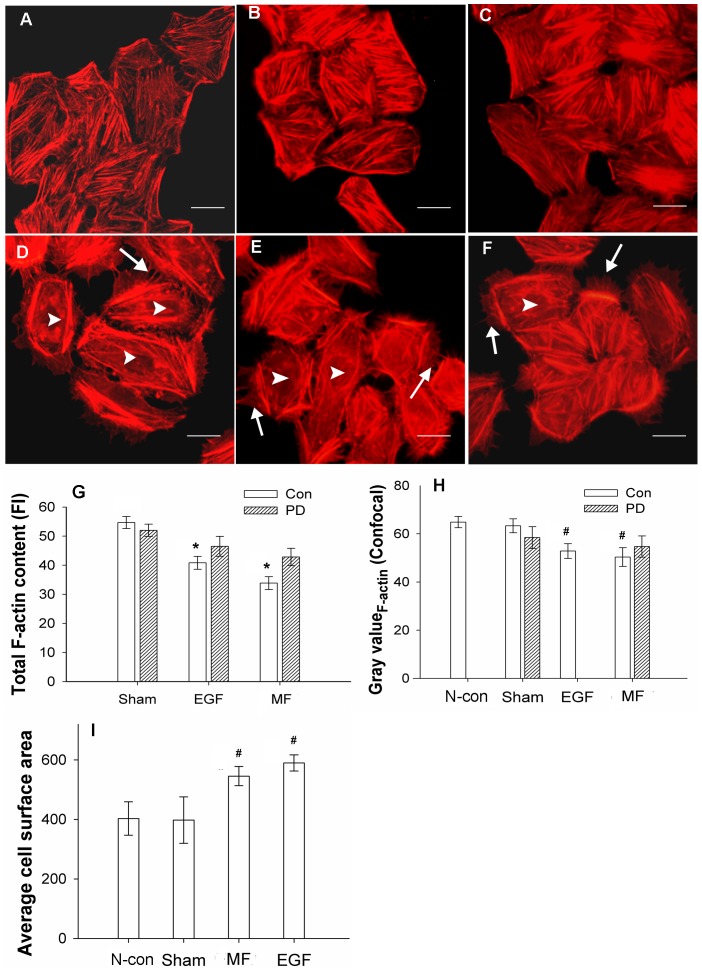
MF induced stress fibers and F-actin content decreases and cell surface increases in FL cells. A: negative control (N-con); B: sham-exposed; C: pre-treated with 1 µM PD (+PD); D: pre-treated with 100 nM EGF (EGF); E: exposed to 0.4 mT power frequency MF (MF); F: pre-treated with 1 µM PD, then exposed to MF (+PD+MF). Microfilaments above were labeled with phalloidin-TRITC and photographed with an Olympus BX51 immuno-fluorescence microscope (×400). G: Decreases in total F-actin content in EGF- and MF-treated cells, measured by flow cytometry, P<0.05 (*). H: Gray value summary of F-actin content of FL cells from confocal images by ImageJ analysis, compared with N-con and Sham, P<0.01 (#). I: Average cell surface area increased, results were analyzed by ImageJ; compared with N-con and Sham, P<0.01 (#). Arrow: newly grown filopodia, arrowhead: loss of stress fibers in central area of the cell. Bar in A–F: 10 µm. The lines on each bar in G–I stand for standard deviation (SD), and the detailed information of experimental conditions and repeating numbers of samples is seen in Table 1.

**Figure 3:**
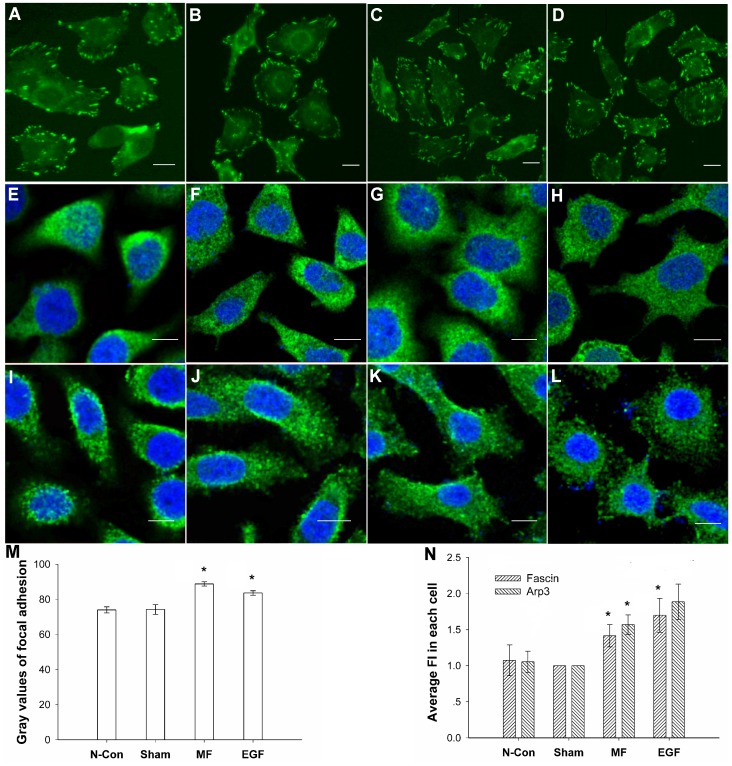
Increases in vinculin-associated focal adhesions in FL cells induced by power frequency MF. Vinculin stained: A–D; fascin stained: E–H; Arp3 stained: I–L. A: negative control (N-con); B: sham-exposed; C: exposed to 0.4 mT power frequency MF (MF); D: pre-treated with 100 nM EGF (EGF); E: negative control (N-con); F: sham-exposed; G: exposed to 0.4 mT power frequency MF (MF); H: pre-treated with 100 nM EGF (EGF); I: negative control (N-con); J: sham-exposed; K: exposed to 0.4 mT power frequency MF; L: pre-treated with 100 nM EGF (EGF)); M: summary of the gray value analysis of the proportion of vinculin-associated adhesion spots to the total cell area, analyzed by ImageJ; compared with N-con and Sham P<0.05 (*); N: summary of the gray value analysis of fascin and Arp3 by ImageJ analysis, compared with N-con and Sham P<0.05 (*). Bar in A–L: 10 µm. The detailed information of experimental conditions and repeating numbers of samples is seen in Table 1.

After exposure to a 0.4-mT power frequency MF for 30 min, FL cells grew many new filopodia that had a distinctly more spine-like structure (arrow), with new lamellipodia in the spreading front (arrow head, Fig. 1D and Fig. 2E) and more vinculin-associated focal adhesions at the leading edge of the cell with a 19.6% increase in the gray value, as analyzed by the ImageJ program (Fig. 3C and 3M, ImageJ 1.46 developed by NIH, Bethesda, Maryland, USA), similar to the effects of EGF (Fig. 1C, 2D, 3D and 3M), for which there was a 12.6% increase. In addition, the cells possessed flatter cellular shapes (Fig. 1D and Fig. 2E) under exposure. Gray value analysis of cell surface area calculations by software ImageJ (Fig. 2I) showed a 37.21% increase in the average surface area of cells under the MF conditions compared to those of the sham, which was similar to the48.31% increase in EGF groups (Fig. 1C, Fig. 2D and 2I).

**Figure 4:**
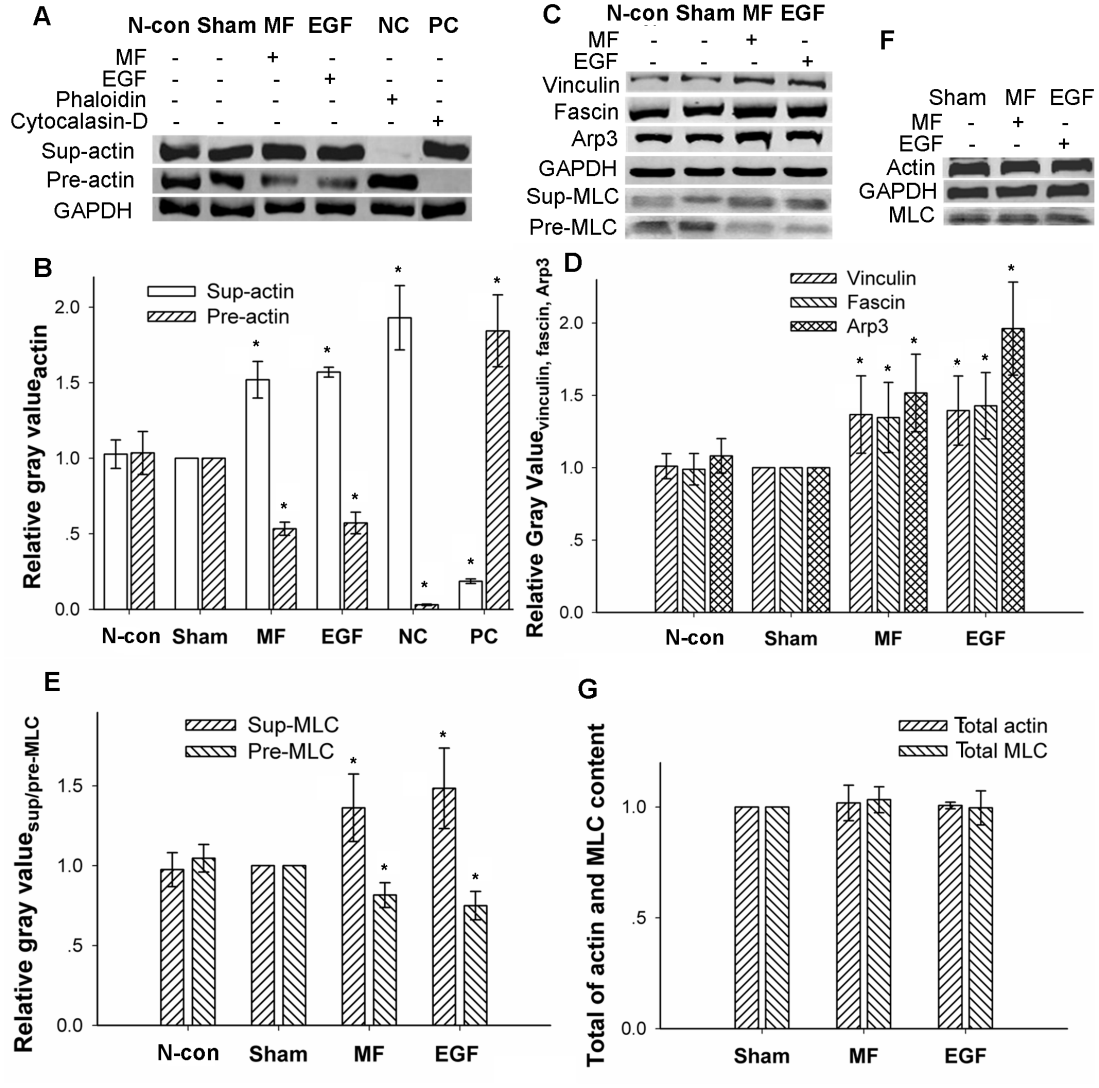
Motility-associated protein content in FL cells affected by power frequency MF by WB assays. A: F-actin/G-actin content in supernatant or precipitate; NC: negative control, 1 µM phalloidin was added when cracking the cells; PC: positive control, 1 µM cytochalasin-D was added when cracking the cells. B: gray value summary of actin content by software ImageJ, compared with N-con and Sham P<0.05 (*). C: focal adhesion-associated signal protein vinculin, filopodia-associated signal protein fascin, lamellipodia-associated signal protein Arp3, and stress fiber-associated signal protein MLC content. D: gray value summary of vinculin, fascin, and Arp3 by software ImageJ, compared with N-con and Sham P<0.05 (*). E: gray value summary of stress fiber-associated MLC by software ImageJ, compared with N-con and Sham P<0.05 (*). F: total content of actin and MLC in FL cells, compared with N-con and Sham. G: gray value of total content of actin and MLC protein in FL cells; compared with Sham P>0.05. The detailed information of experimental conditions and repeating numbers of samples is seen in Table 1.

Furthermore, a decrease in the dye fluorescence intensity (FI) of the total F-actin content (Fig. 2G) from 54.7±2.1 (mean±SD, and the same as the rest) to 33.8±2.2was found by flow cytometry assays, indicating a 38.21% decrease. This decrease was very similar to the effect of EGF on the total F-actin content (40.8±2.1) of the cell (Fig. 2G), which resulted in a 25.41% decrease (all F-actin content values were measured in TFC units). Similar results were observed by confocal microscopy. When analyzed by software ImageJ, a loss of stress fibers in the central area of the cell was observed (arrowhead in Fig. 2D–F), with a reduction from 63.33±2.91 to 50.34±3.90 for a 20.52% decrease with the MF exposure (Fig. 2E and 2H) and a decrease to 52.86±3.04 with EGF treatment for a 16.54% reduction (Fig. 2D and 2H). Similar results were also obtained with Western blots assays (WB), in which F-actin was separated from free G-actin through ultra-high-speed centrifugation. It was observed that the total content of F-actin, compared with the sham exposure group, decreased by 46.64±4.37% (MF) or 42.85±7.14% (EGF) (Fig. 4A and 4B), while free G-actin content increased by 52.00±12.13% (MF) or 57.05±3.25% (EGF), resulting in a F/G-actin ratio that reduced by 64.89% (MF) or 63.61% (EGF) compared with sham-exposed cells (Fig. 4A and 4B) (all calculations of percent changes for gray values analyzed by ImageJ in the article are summarized in the material and methods). Morphological responses, including newly formed protrusion patterns, decreases in total F-actin content and the F/G-actin ratio, and increases in vinculin-associated focal adhesion spots in the leading edge were similar to those in EGF-treated cells (Fig. 1C, Fig. 2D, Fig. 3D, and Fig. 4A–D).

However, the total content of actin in cells was not affected by the MF. As shown in Fig. 4F and Fig. 4G, it was observed that the total content of actin in FL cells in the MF- and EGF-treated groups was almost the unaffected as that in sham-exposed cells, suggesting that the MF and EGF treatment only influence the ratio of F/G-actin. Information of detailed sample size is shown Table 1.

**Table 1:** The information of experimental conditions and repeating numbers of samples for each target.

	Condition	Number of trials (m)	Number of parallels (n)	Number of analyzed cells
**AFM (** **Fig. 5** **)**	Each condition	4	4	–
**SEM (** **Fig. 1** **)**	N-con	3	6	10
	+PD+MF	3	6	12
	Rest	6	12	34
**Focal adhesion spots/fascin/Arp3 (confocal, ** **Fig. 3** **)**	N-con	3/3/3	12/6/7	29/24/19
	Sham	4/3/3	12 6/6	43/27/25
	+MF	4/3/3	12/6/5	37/26/24
	+EGF	3/3/3	12/5/6	32/22/25
**F-actin (confocal, ** **Fig. 2** **)**	N-con/Sham	3	6/10	35/66
	+MF/+EGF/+PD/+PD+MF	5	10	48/41/37/43
**Cell area (confocal, ** **Fig. 2** **)**	N-con	3	6	33
	Sham	3	6	72
	+MF/+EGF	3	6	51/45
**F-actin (flow cytometry, ** **Fig. 2** **)**	Sham	6	11	∼5.5×10^5^
	+MF	5	9	∼4.5×10^5^
	Rest	3	6	∼3×10^5^
**F/G-actin WB (** **Fig. 4** **)**	NC/PC	9	16	∼6×10^6^
	Rest	15	30	∼10×10^6^
**Vinculin WB (** **Fig. 4** **)**	Sham	5	9	∼4×10^6^
	Rest	4	7	∼3×10^6^
**Fascin WB (** **Fig. 4** **)**	Each condition	4	8	∼3×10^6^
**Arp3 WB (** **Fig. 4** **)**	Each condition	3	6	∼2×10^6^
**MLC WB (** **Fig. 4** **)**	+EGF	4	7	∼3×10^6^
	Rest	5	8	∼4×10^6^
**F-actin content/cell area and recovered (** **Fig. 6** **)**	Sham	3	3	29
	0/0.5/1/1.5/2 h	3	3	24/19/17/15/25
**Recovered content of proteins (** **Fig. 6** **)**	Each condition	3	4	∼2×10^6^
**Recovered content of MLC (** **Fig. 6** **)**	Sham	3	4	∼2×10^6^
	Recovered	4	5	∼3×10^6^
**EGFR ligand release (** **Fig. 7** **)**	PMA	5	5	–
	Control	4	4	–
**Cell migration (** **Fig. 8** **)**	Sham/MF	6	12	–
	EGF/MF+EGF	3	5	–
**[Ca^2+^]_i_ (** **Tab. 1** **)**	Sham	6	30	∼3×10^6^
	Rest	3	10	∼1×10^6^

∼: about.

m: the number of independent experimental trials carried out for each biological target.

n: the number of total parallel samples.

Compared with the results of negative control group (N-con), in sham-exposed cells present no significant difference in morphology (Fig. 1A and 1B; Fig. 2A and 2B), F/G-actin ration (Fig. 2A, 2B and 2H; Fig. 4A and 4B), related signaling proteins by confocal (Fig. 3A, 3B,3E, 3F, 3I, 3J, 3M and 3N) or by WB (Fig. 4C–E) between the control cells and the sham-exposed cells.

### 2. Power Frequency MF Reduces the Efficiency of Microfilament Formation in vitro

**Figure 5:**
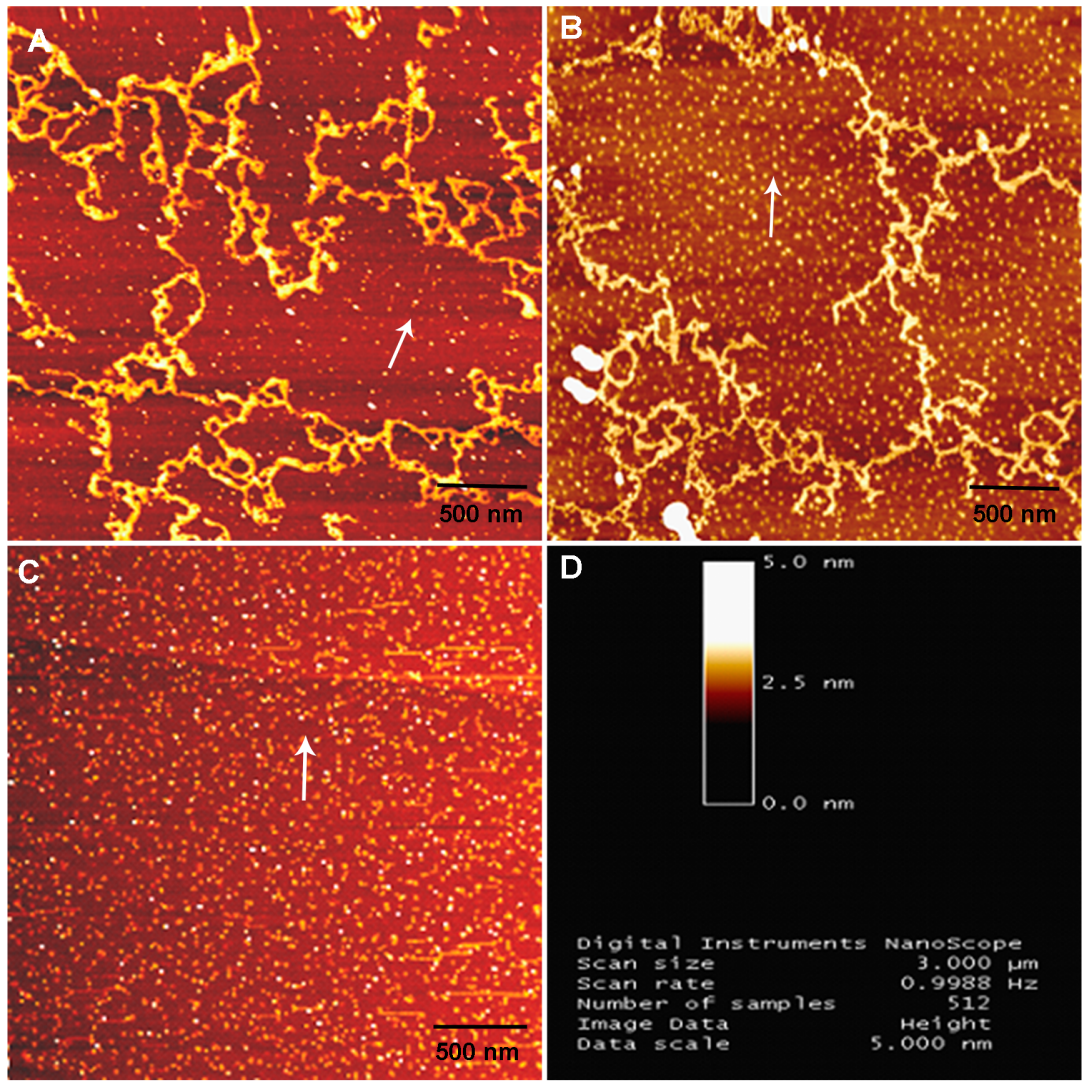
Decrease in the efficiency of F-actin assembly by power frequency MF. A: sham-exposed G-actin to power frequency MF observed by AFM. B: exposed G-actin of self-assembled microfilaments to power frequency MF observed by AFM. C: free G-actin, without self-assembly, in sham exposure observed by AFM. D: AFM image index information. Arrow: free G-actin. Bar scale at the bottom of the image: 500 nm. The detailed information of experimental conditions and repeating numbers of samples is seen in Table 1.

Cytoskeletal microfilaments are electronic polarized and are potential targets of power frequency MF exposure [25]–[26], [28]–[29]. We found that power frequency MF exposure affected microfilament formation at a molecular level in vitro. In the presence of ATP, G-actin monomers self-assembled to form microfilament fibers (branch-like structures in Fig. 5B) in vitro, which were then examined by AFM. The results show that the actin inboth the sham and exposed groups presented well-formed branch-like actin filaments. However, there were significantly fewer filaments in the MF-exposed samples than in the sham-exposed samples, with much more free G-actin (arrow, light dots against the background in Fig. 5B), indicating a decrease in the efficiency of F-actin assembly. This result may partially explain the decrease in total cellular F-actin content with a smaller ratio of F/G-actin and fewer cellular central stress fibers in FL cells observed upon the MF exposure.

### 3. Power Frequency MF Affects the Protein Contents of Significant EGFR-cytoskeleton Downstream Signaling Molecules

It was shown that blocking EGFR activation with PD contributed to recovery from power frequency MF-induced EGFR clustering [24] and may partially recover the cytoskeletal reorganization. It was suspected that the MF could activate EGFR to stimulate the activation of downstream signaling molecules, some of which participate in cytoskeletal reorganization and motility. Through WB assays, we examined the protein content of several relative EGFR downstream signaling molecules, such as fascin, Arp3, vinculin and MLC, which tightly associate with the cytoskeleton and are involved in motility.

It was found that fascin, Arp3, and vinculin were highly soluble and were only present in the supernatant after ultra-high-speed centrifugation. The gray values of the WB protein bands were analyzed and calculated by the software ImageJ. The results showed that, compared with the sham-exposed cells, in which the relative level of protein content was as 1 or 100%compared with itself (Western bloting bonds seen in Fig. 4, and cytoskeleton gray values, seen in Fig. 2) analyzed by software ImageJ, the protein content of fascin, Arp3 and vinculin, which trigger the formation of filopodia, lamellipodia, and associated focal adhesions, in the exposed cells, increased by 34.7±21.2%, 51.7±22.2% and 36.8±21.7% (Fig. 4C and 4D), respectively. Furthermore, this trend was highly similar to that for EGF treatment, which resulted in increases of 42.9±22.8%, 96.2±32.1%, and 39.5±20.9% (Fig. 4C and 4D), respectively.

Similar results were obtained from the results generated from the confocal experiments. When exposed to MF, the mean of total FI was 1342.9±114.1 (Fig. 3G) in fascin-stained cell, or 1473.7±147.3 (Fig. 3K) in Arp3-strained cells, which was 41.6±13.4% or 56.9±12.4% higher than that of the sham-exposed (953.5±55.2 in fascin-stained, Fig. 3F; or 929.0±126.3 in Arp3-stained, Fig. 3J), respectively. However, the distributions and intensities of FI signal of the exposed cells were different from those in the sham-exposed groups. In both exposed samples, it can be seen that higher intensities of FI stained targeted proteins concentrated surrounding the nucleus, while with larger cell surface areas and spreading edges which are similar to the data of the flatter cell shapes (Fig. 2E and 2I). Again, in the cells of the MF exposed groups, fascin and Arp3 were found in the new grown spine-like protrusion structures (Fig. 3G and 3M), indicating the field induced re-distribution and re-locating of these two signal proteins to support cell migration or invasive functions. EGF treated cells (Fig. 3H, 3L and 3N) showed similar effects as the MF exposed cells.

However, MLC, which binds to stress fibers and is an indicator of the amount of stress fibers present [13], was found both in the supernatant and the precipitate. The MLC in the precipitate was bound to F-actin to form stress fibers, while that in the supernatant was free. The protein content was investigated, and we found an increase of 36.282±1.16% in the supernatant (sup-MLC) but a decrease of 18.39±7.75% in the precipitate (pre-MLC) (Fig. 4C and 4E) in the exposed group compared with Sham, indicating a decrease in total stress fibers. Similar results were also obtained in the EGF-treated group, for which an increase of 48.46±25.19% in the supernatant and a reduction of 25.19±8.90% in the precipitate was observed (Fig. 4C and 4E). These results indicate that power frequency MF indeed affects motility-related cytoskeletal reorganization through relevant signaling pathway molecules. Similarly, the effects of the MF on total actin content and total MLC content in both the MF- and EGF-treated groups were found to be quite conserved compared to the sham-exposed group (Fig. 4F and 4G). Information of detailed sample size is shown Table 1.

### 4. Power Frequency MF-induce Transient Morphological and Molecular Changes Disappear in 2 Hours after Power Frequency MF is Withdrawn

**Figure 6:**
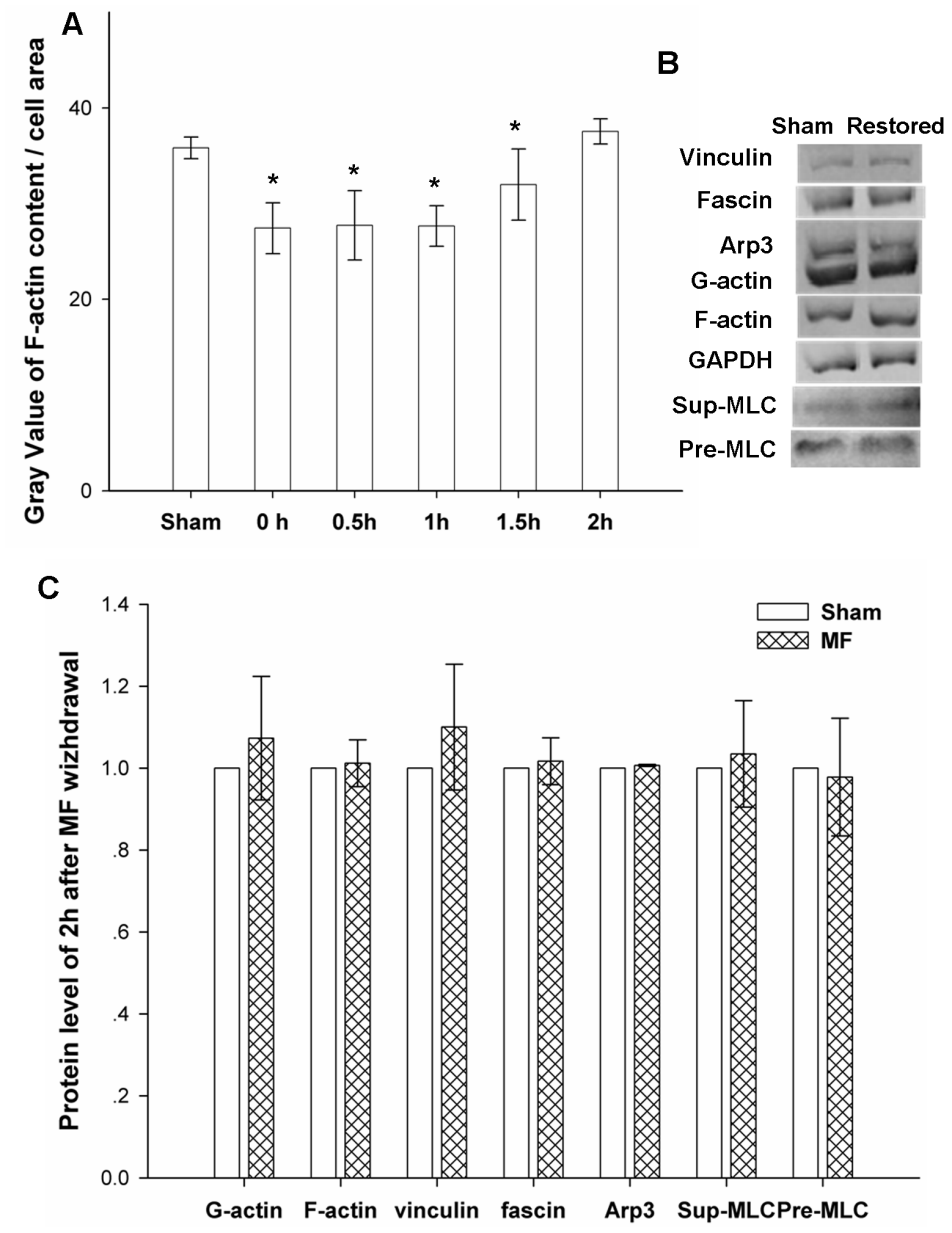
The influence of power frequency MF was greatly reducedby 2hours after withdrawal of the field. A: Time-dependent recovery of F-actin content after withdrawing power frequency MFs. Sham: sham-exposed to power frequency MF for 0.5 hours; 0–2 h: exposed to power frequency MF for 0.5hours,field withdrawn and then sham-exposed for 0hour to 2hours; the summary of the gray value analysis of F-actin and cell area was analyzed by software ImageJ; compared with Sham, 0–1.5 h P<0.05 (*), 2 h P>0.05. B: content of vinculin, fascin, MLC and Arp3 proteins in FL cells 2 hours after withdrawing power frequency MF; C: gray value summary of vinculin, fascin, MLC and Arp3 content in FL cells 2 hours after exposing to MFs by software ImageJ, compared with Sham, P>0.05. The detailed information of experimental conditions and repeating numbers of samples is seen in Table 1.

Cells were prepared and treated following the same protocols for exposure as indicated, and then, the power frequency MF was withdrawn. The resultant samples were incubated with 5% CO_2_ at 37°C for up to 2 hours. The resultant effects on cell or molecular levels are shown in Figure 6A, 6B and 6C, respectively. It was found that withdrawal of the field resulted in a total recovery from field exposure-induced changes at the cell morphology and molecular levels, indicating that the influence of the MF is not likely permanent but is, more likely, recoverable. Information of detailed sample size is shown Table 1.

### 5. Power Frequency MF Downgrades the Release of EGFR Ligands

To address if the invasive structural transitions induced by power frequency MF are due to increased EGFR ligand release into the cell cytoplasm membrane stimulated by the field, we carried out assays to investigate the levels of TGF-αligand release in the FL cell line.

**Figure 7:**
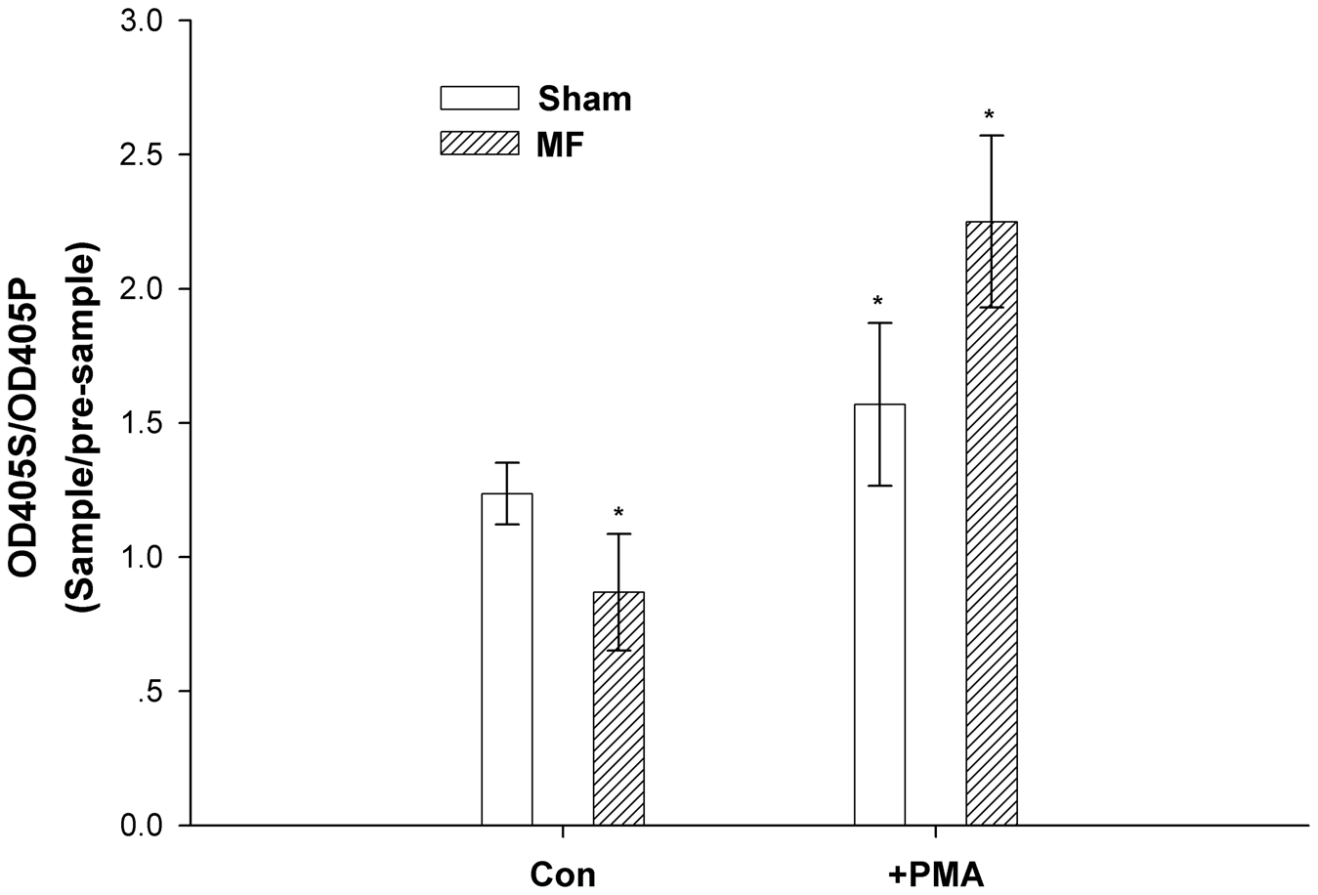
Power frequency MF downgrades EGFR ligand release. FL cells transfected by TGF-α were grouped into 4 conditions: sham exposure, treated with PMA as a positive control, 0.4 mT power frequency MF exposure, and treated with PMA plus power frequency MF exposure. The cells were washed, and conditional medium was added. After 30 min, the medium from each group was collected as the corresponding pre-sample. Then, medium was collected again for all groups after incubation in the indicated conditions for 30 min. OD data were measured for each sample or pre-sample. The data presented in Figure 7 are the ratio of the OD of sample (OD405S) over the OD of pre-sample (OD405P). P<0.05 (*) between each two group. The detailed information of experimental conditions and repeating numbers of samples is seen in Table 1.

Power frequency MF induces EGFR self-oligomerization [24], and this effect is independent of EGFR ligands under constitutive conditions, as we observed that the constitutive shedding of TGF-αalpha was inhibited by the MF with a 29.75% decrease in the OD405S/405P ratio, from 1.24±0.12 to 0.87±0.22 (Fig. 7). However, when other stimuli, such as PMA (phorbol 12-myristate 13-acetate, an activator of PKC), were present, the induced shedding of TGF-αwas dramatically increased by 43.41%, from 1.57±0.30 to 2.25±0.32, by the MF (Fig. 7). It is very likely that the MF further enhances the phosphorylation effect caused by PMA, which is a PKC activator and induced an increase in the shedding of TGF-αby 26.90%, from 1.24±0.12 to 1.57±0.30. However, whether the MF also enhances the overall phosphorylation in EGFR signaling pathways requires further investigation.

In Fig. 7, the data show that ligand production was inhibited by exposure to a 0.4 mT power frequency MF, indicating that all of the above effects we observed were not simply due to increased EGF production in the EGFR upstream pathway. Information of detailed repeating trails and sample size is shown Table 1.

### 6. Power Frequency MF Upgrades the Average Cell Migration Rate

The power frequency MF induced a pre-mobile state in FL cells, with more filopodia and lamellipodia around cell periphery and less stress fibers in the cell center, and simultaneously activated the cytoskeleton-related EGFR signaling pathway. We then explored whether the motility of FL cells was affected by the MF.

**Figure 8:**
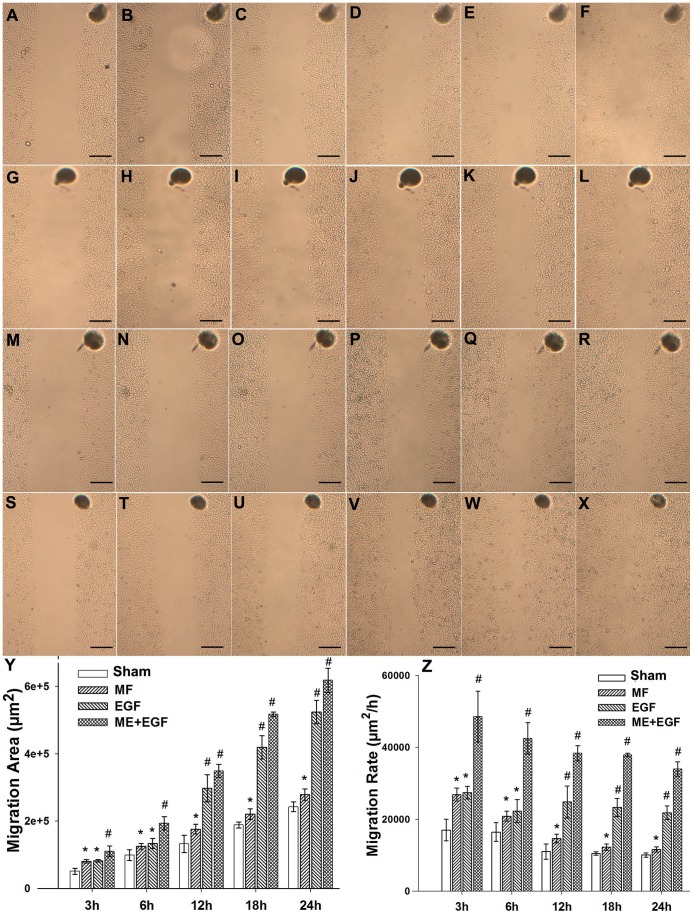
Influence of power frequency MF on the average migration rate of FL cells. A–F: Sham for 0 h, 3 h, 6 h, 12 h, 18 h, and 24 h successively; G–L: MF for 0 h, 3 h, 6 h, 12 h, 18 h, and 24 h successively; M–R: EGF for 0 h, 3 h, 6 h, 12 h, 18 h, and 24 h successively; S–X: MF+EGF for 0 h, 3 h, 6 h, 12 h, 18 h, and 24 h successively; Y: Effects on FL cell migration area; the areas were calculated by software ImageJ; compared with Sham, P<0.05(*) or <0.01(#); Z: Effect on the migration rate of FL cells, compared with Sham, p<0.05 (*) or <0.01(#). Bar: 200 µm. The repeating and experimental condition information is seen in Table 1.

The results are shown in Fig. 8. It was observed that, with the MF exposure, cells became more mobile, and the average migrated areas (×10^3^ µm^2^) increased from 51.07±9.07, 98.84±15.81, 132.46±25.42, 188.53±8.59, and 242.34±14.45 in the sham group, to 80.70±5.32, 125.28±8.28, 176.17±14.48, 220.97±15.84, and 278.44±16.99 in the MF exposed group, for 3, 6, 12, 18 and 24 hours (Fig. 8A–L, and 8Y), respectively. Meanwhile, the average migration rates (×10^3^ µm^2^/h) increased from 17.02±3.02, 16.47±2.63, 11.04±2.12, 10.47±0.47, and 10.09±0.60 in Sham to 26.89±1.77, 20.88±1.38, 14.68±1.20, 12.27±0.87, and 11.60±0.70 in exposed for 3, 6, 12, 18 and 24 hours (Fig. 8A–L, and 8Z), respectively. The EGF treatment alone firstly had a similar effect on the mobility of the migration compared with the MF treated cells in the first 6 hours, then followed by a remarkable impact on accelerating the cell migration in longer treatments. The elevated migration areas (×10^3^ µm^2^) were 82.26±4.10, 133.71±14.60, 297.71±40.39, 419.21±34.67, and 523.73±34.40 (Fig. 8M–R, and 8Y); and the corresponding increased migration rates (×10^3^ µm^2^/h) were 27.42±1.80, 22.28±3.73, 24.80±4.44, 23.28±2.54, and 21.82±1.89, for 3, 6, 12, 18 and 24 hours (Fig. 8M–R, and 8Z), respectively. We noted that in MF plus EGF treated groups (MF+EGF), the enhancements on the migration rates were even much more significant, and the increased migration area (×10^3^ µm^2^) was 110.48±16.01, 193.36±19.82, 349.16±19.37, 517.19±6.56, or 618.04±36.12 (Fig. 8S–X, and 8Y), with the corresponding increased migration rate (×10^3^ µm^2^/h) at 48.61±7.04, 42.54±38.40, 38.40±2.13, 37.92±0.48, or 33.99±1.98 with the MF+EGF treatment for 3, 6, 12, 18 or 24 hours (Fig. 8S–X, and 8Z), respectively. The groups without field exposure (N-CON) had no significant difference with the sham group (data not shown here).

The factor of larger migrated areas and higher average migration rates of MF exposure groups at the corresponding exposure lengths suggests that the MF exposure is likely to elevate the mobile capacity of FL cells even in the absence of EGF, indicating that the power frequency MF possibly invokes the motility mechanism in certain degree. The influence of the MF similar to that of EGF especially in the first 6 hours, may suggest the possibilities of MF may play the role of EGF to activate cell migration, and of that a possible MF-activating-motility mechanism is likely to involve in the EGF pathway.

With increase in the treating time, the migration rates in all groups presented a slow-down trend. It’s speculated that it was due to decrease in the migration stress with the time passed by. When more and more cells migrate to the blank area, the cell density along the borders decreases gradually; the cells in the mitigating front feel less migration stress from the neighboring bulk cells. Further more, compared with the groups with EGF in the presence (EGF and EGF+MF) which shows a stronger or more durable effect on cell migration, the MF group, has a lower migration rate and a higher migration rate reduction gradient, indicating the MF alone affects cell migration in a way of short term or transiently, which agrees with our previous results. It may also suggest, the cells becomes more and more accustomed to MF exposure in the prolonging the exposure time. Additionally, EGF and MF is likely to have an additive effect on the cell migration rate (Fig. 8Z).

## Discussion

The actin cytoskeleton, a main component of the cytoskeleton, plays a significant role in cell motility. In a migrating cell, actin microfilaments in the spreading leading edge are usually denser than those in the resting cells so that their motility and chemotaxis functions can be achieved. A migrating cell grows more invasive protrusion structures, such as filopodia and lamellipodia, with more focal adhesions in the leading edge than in resting cells, resulting in flat cell shapes with altered cell morphology. It is also clear that cells undergoing tumorigenesis have been observed to have altered cytoskeletons with enhanced motility and less polarity [30]–[31]. Similar effects were also observed in migrating cells during many biological processes, such as tissue repair and regeneration [1], [2], [18], [19]. All of these cytoskeletal dynamics of migrating cells are highly associated with the EGFR signal pathway because there is intensive cross talk between the cytoskeleton and the EGFR signal pathways.

Power frequency MF serves as an extracellular stimulating factor to trigger cells. It is proposed that power frequency MF-induced changes in the cytoskeleton are due to the presence of field-cell interaction sites at the plasma membrane level in different cell types [22]–[23], [25]–[26], [29], [32]. Our earlier work showed the formation of PD-prevented EGFR-clustering induced by a power frequency MF [24] in the absence of EGF stimulation, suggesting that EGFR is a membrane target for power frequency MF interaction, which disrupts the EGFR signaling pathway in a manner that involves receptor oligomerization. It also suggests that the effect of power frequency MF acting on EGFR clustering is somehow similar to that resulting from EGF stimulation. However, whether power frequency MF also activates EGFR signaling and evokes the downstream signal phenomena remains unclear.

Our previous work also revealed that power frequency MF exposure resulted in altered cytoskeleton morphology in FL and CHL cell lines [22]–[23]. Understanding how power frequency MFs affect cell cytoskeleton-associated motility is very important for studies of the safety of power frequency MF exposure to humans and for potential clinical applications.

This study is part of a continuous effort to understand the effects of power frequency MF-induced cytoskeleton changes associated with the EGFR signal pathway, showing that exposure of cells to 0.4 mTpower frequency MF induces FL cells to shift to a migration-like state at the morphological and molecular level. Detailed studies have revealed that, in the absence of EGF, sham-exposed cells showed no difference from the negative control cells (Fig. 1A and 1B; Fig. 2A, 2B, 2H and 2I; Fig. 3A, 3B, 3E, 3F, 3I, 3J, 3M and 3N; Fig. 4A–E), indicating that the switch-off exposure system had no contribution to the bio-effects of the MF on FL cells, while the field-exposed cells appear to have distinct invasive characters at the morphological and molecular levels, including great increases in filopodia, lamellipodia (Fig. 1D and Fig. 2E), and focal adhesions (Fig. 3C) in the spreading edges of cells and a weaker central F-actin cytoskeleton (Fig. 2E).It has also been found that there is an overall decrease in F-actin content (Fig. 2G and 2H) and a decrease in the F-actin/G-actin ratio of whole cells (Fig. 4B). The increased filopodia are accompanied by a 34.69% increase in the filopodia-associated signal protein fascin (Fig. 4D), while the increased lamellipodia are accompanied by a 51.67% increase in the lamellipodia-associated signal protein Arp3 (Fig. 4D). Similar patterns are observed for the focal adhesion-binding protein vinculin (Fig. 4D), which increases by 36.74%, compared with the sham groups. From these results, it is proposed that power frequency MF activates EGFR-associated cytoskeleton signal pathways (as shown in “regulations of actin dynamic” on Cell Signaling website, http://www.cellsignal.com/reference/pathway/Regulation_Actin.html), which leads to the formation of protrusional structures.

**Figure 9:**
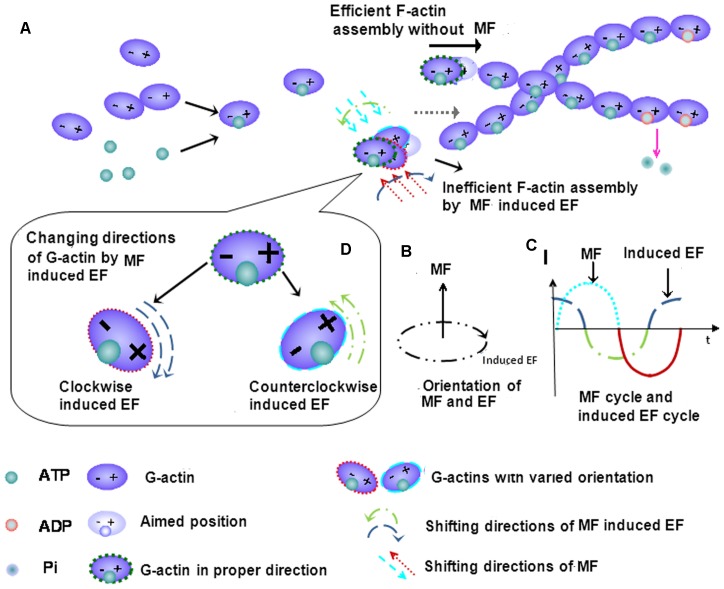
A possible model for power frequency MF interaction with the actin cytoskeleton. A proposed physics model of power frequency MF disrupting F-actin assembly. The actin monomers can be seen as electric dipoles, and it is necessary for the monomers to be in a proper angle and orientation to join to the F-actin string (A). It’s suggested that power frequency MF induces electrical fields that have an effect on actin monomers. In the center of the magnetic field generation device, the direction of MFs is vertical to the coil plates, and the induced electrical fields (EFs) are parallel to the plates in clockwise or counterclockwise orientation (B). The intensity of the MF is vibrated as a sine function at the frequency of 50 Hz (C). Then, the intensity of the induced EF changes as a cosine function (C). Thus, in the MF-induced EF, the orientation or/and position of the actin dipoles flips over and over, following the EF direction, as shown in B (D). While the mean time for an actin monomer binding to F-actin is approximately 0.02 seconds, the induced EF changes its direction before the other candidate monomer with a proper orientation binds to the F-actin stream. As the result, the efficiency of F-actin assembly decreases.

To understand the reason for the decreased F-actin content and assembling efficiency induced by the power frequency MF exposure, we attempt to provide an explanation. Firstly, at the cellular level, it has also been shown that stress fiber content decreases in association with decreased levels of the stress fiber-associated protein MLC, which binds with F-actin fibers to form stress fibers and maintain cell shape and contractibility [10]–[13]. However, there are increased levels of MLC in the cytosol, which is evidence for a weakened cytoskeleton, and the flat cell morphology. Secondly, it is understood that, in an activated invasive state, a cell supplies more G-actin to its spreading edge than the rest of the cell, resulting in fewer center stress fibers. Furthermore, field exposure likely directly interrupts actin polymerization, especially under conditions where protection from the rest of the cellular contents in the absence (Fig. 5B). To understand the interaction mechanism between power frequency MF and the cytoskeleton, we try to draw a physical picture to explain. As it is known, G-actin moves in the cell and exists as an electric dipole. Exposure to power frequency MF associated with induced electrical fields affects actin monomers via the magnetic force (F_m_), the Lorentz force (F_L_) and the electric force (F_e_). F_m_ and F_L_ are far weaker than F_e_ due to the slow movement of the G-actin in the cell. Therefore, we take F_e_ to analyze the effect of power frequency MF on F-actin assembly. As shown in Fig. 9, it is necessary for a candidate G-actin monomer to be in a proper position and angle to join to the F-actin string (Fig. 9A). However, in the center of the magnetic field generation device, in which cells were exposed, the direction of the magnetic field (MF) is vertical to the coil plates, while the induced electric field (EF) is parallel to the plates in clockwise or counterclockwise orientation (Fig. 9B). Assuming that the MF vibrates as a sine function of 50 Hz, the intensity of the induced EF vibrates as a cosine function (Fig. 9C). The polarized direction and/or position of the actin monomer vibrate with the shifting of the EF direction. Under these conditions, we know that the time for a 50 Hz power frequency MF field to switch field directions is 0.02 sec, and thus the time circle of the induced electrical field in actin is also 0.02 sec, which is coincidental to the average actin assembly time factor of∼0.02 seconds [33] (the average time needed for a free actin monomer to bind to another in an F-actin fiber). In the power frequency MF-induced EF, the orientation and/or position of the actin dipoles flips over and over, following the continuously switching-direction EF orientation induced by power frequency MF (Fig. 9D). The actin electric dipoles are forced to follow the field to continuously change their own direction and/or position and switch back and forth. Under these conditions, it becomes more difficult for the monomers to be stabilized and constructed into F-actin filaments; consequently, actin polymerization becomes even more difficult, which reduces the efficiency of F-actin assembly (Fig. 9).

Actin is known to be conservative in cells. These data suggest that power frequency MF has a disturbing effect that results in the construction of fewer stress fibers so that fewer G-actin monomers are employed in the center area and more free G-actin monomers are disassociated in the local cytosol, as indicated by our result showing the decreased F-actin/G-actin ratio in cells and the decreased efficiency of actin assembly in vitro. However, power frequency MF is likely to activate the motility mechanism to upgrade the actin requirement in the activated leading edge, which leads to the increased growth of new motility organelles around the outer ring of the cell. We also observed that the mean total F-actin content decreases in the exposed samples, which may be interpreted to mean that the increase in the amount of F-actin for the assembly of invasive structures cannot balance the decrease in F-actin from the central area of the cell because stress fibers are the biggest consumer of G-actin. These results suggest that, firstly, power frequency MF alone directly contributes to a certain degree to the decrease in assembly efficiency; and, secondly, the activation of cellular motility causes additional decreases in the effect that low actin assembly exerts on central stress fibers. The process also involves the increased levels of the signal proteins of fascin, Arp and vinculin, indicating the activation of the EGFR-cytoskeleton signaling pathways in preparation for migration.

It is suspicious that the above effects induced by power frequency MF exposure may be a result of the field inducing more ligands to activate the signal pathways. However, because increased intracellular EGFR ligand production by field exposure does not occur under these conditions (Fig. 7), the possibility that the field induces more ligand production to stimulate the cytoskeleton transition can be excluded.

Our findings in this study showed that blocking EGFR activation with PD did not completely prevent all power frequency MF-induced growth of new filopodia, lamellipodia and focal adhesions and did not reverse the loss of central stress fibers (Fig. 1E and Fig. 2F), indicating that PD-sensitive EGFR is not the sole contributor to power frequency MF-induced cytoskeletal reorganization.

Our results also suggest the involvement of Ca^2+^mobilization in power frequency MF-induced cytoskeleton effects. It is widely accepted that EGF binding to EGFR evokes a rapid enhancement ofCa^2+^influx through the membrane ion channels, inducing several Ca^2+^-related effects on cytoskeletal functions [34]–[37]. An optimal [Ca^2+^]_i_ is required to regulate dynamic cellular functions and ABP activity [38]–[39]. A large amount of evidence has accumulated to show that electromagnetic fields (EMFs), including power frequency MF, evoke Ca^2+^influx, induce EGFR oligomerization [40]–[41], and have an influence directly or indirectly on fascin [42], Arp2/3 [43], MLC [44], and vinculin [45], which strongly suggests that [Ca^2+^]_i_ also plays an important part in power frequency MF-induced cytoskeleton reorganization. Furthermore, our data show that power frequency MF elevated the cytosol Ca^2+^ level (Table 2), which is similar to reported results [46]–[49]. These results suggest that power frequency MF may act through membrane proteins, such as growth factor receptors and Ca^2+^ channels, to activate relevant signals and evoke the cell transition to a migration-like state; further research efforts are required to improve our understanding. A 30-min exposure to a 0.4 mT power frequency MF is likely too brief and weak to induce any long-term cellular modifications. The results from the recovery experiments show that the exposure likely has short-term effects (Fig. 6A–C). This may be good news for people who are exposed to a short-term power frequency MF in their daily lives. However, it is not clear whether a 30 min or longer exposure to power frequency MF would cause any epigenetic consequences and other accumulation effects on the cell, which still needs our concerns.

**Table 2:** [Ca^2+^]_i_ in FL cells (FI_526_).

	Sham	EGF	MF
**Mean**	30.55	38.34	39.87
**Error (SD)**	1.04	1.53	4.09

After fixed in 4% paraformaldehyde at 4°C overnight, FL cells were stained with 24.8 µg/ml Fluo-3/AM(EX/EM: 488 nm/525 nm) for 40 min at 37°C in the dark. A 0.5-ml (1×10^5^/ml) cell suspension was collected for each group for flow cytometry measurements. The detailed information of experimental conditions and repeating numbers of samples is seen in Table 1.

From the results mentioned above, we conclude that (1) power frequency MF activates the EGFR-initialed cytoskeleton signaling pathway and evokes a series of cytoskeletal responses to the field stimulus in a manner similar to EGF activation; (2) as an electrical dipole, G-actin assembly into F-actin is likely directly disrupted by power frequency MF exposure; (3) the MF activates the cell motility and relative mechanism; (4) the 0.4 mT 30 min exposure likely induces acute effects on the cell motility. The power frequency MF alone affects cell motility in a way of short term or transiently; and (5) the membrane protein EGFR is a potential target of the MF exposure, which may be tightly associated with other membrane proteins such Ca^2+^ channels during power frequency MF-induced activation. Therefore, we propose the power frequency MF induced motility-cytoskeleton effects are likely due to the fact that power frequency MF works, in a short-term way, similarly to the EGFR ligands to activate the EGFR and then evokes the EGFR downstream signal pathways, to activate Ca^2+^ mobilization, to interrupt cytoskeleton construction, and to activates cell migration. The associated motility signal or functional molecules fascin, Arp3, vinculin and, MLC, which play considerable roles in the assembly and arrangement of F-actin in the activated cytoskeleton, are activated as downstream events to finally cause the cell to transfer to an alert state for invasion-motility.

Our present work sheds a light on our understanding of the possible mechanism of power frequency MF interruption of the motility of FL cells, which may be valuable for drafting safety guidelines for power frequency MF exposure, and for the importance of cell migration and for clinical applications involving wound and tissue repair. The study indicates that power frequency MF may have transient impacts on cell motility, but it is still not known whether it has an accumulating effect on living organisms, which may be critical for those suffering serious diseases. For these purposes, there is more work that needs to be performed in order for us to understand the mechanism of power frequency MF interaction with cells to determine if power frequency MF effects accumulate over longer time spans, if the frequency of MF is critical for the observed effects, and if power frequency MF affects tumor cell migration. At the cellular level, further effort is also needed to determine if the Ca^2+^ channels and inner pools are involved in power frequency MF-induced effects. Additionally, it is still unclear whether power frequency MF affects an other component of the cytoskeleton: microtubules. At the molecular level, we also need more understanding of the interactions between power frequency MF and ligand-acceptor binding in the cell membrane. Further work is required to answer these questions.

## Materials and Methods

### 1. Reagents

EGF (Cat. No: E9644), phalloidin-TRITC (Cat. No: P1951), actin (Cat. No: A3653), adenosine 5′-triphosphate desmodium (ATP, Cat. No: A1852), and dithiothreitol (DTT, Cat. No: D9779) were purchased from Sigma Chemical Co. (St. Louis, MO). PD153035 (PD) was purchased from Calbiochem (EMD Biosciences Inc., Darmstadt, Germany; Cat. No: 234490). Fluo-3/AM (Cat. No: F1242) and Fluo-4/AM (Cat. No: F14201) were from Molecular Probes (Eugene, OR), the G-actin/F-actinIn Vivo Assay Kit was from Cytoskeleton (Cat. No: BK037), andthe SDS-PAGE kit (Cat.No: P0012A), sample buffer (Cat.No: P0015), actin antibody (Cat.No: AA128) and GAPDH antibody (Cat.No: AG019) were from Beyotime. The mouse monoclonal antibody to fascin (Cat.No: ab78487), anti-myosin light chain antibody [MLC] (Cat.No: ab97891), and anti-Arp3 antibody (Cat.No: ab56817) were from Abcam, and the vinculin antibody (Cat.No: 4650) was from Cell Signaling Technology. The secondary antibodies IRDye 800CW goat anti-mouse IgG (H+L) (Cat.No: 926-32210) and IRDye® 680RD goat anti-rabbit IgG (H+L) (Cat.No: 926-68071) were purchased from LI-COR, and anti-rabbit IgG Fab2 Alexa Fluor(R) 488 Molecular Probes (Cat.No: 4412S) was from Cell Signaling. Fluo-3/AM was purchased from Molecular Probes (Eugene, OR) (Cat. No: F1242).

### 2. Cell Preparation and Power Frequency MF Exposure

FL cells were cultured in Minimum Essential Medium (MEM), which was supplemented with 10% heat-inactivated BSA (Gibco, USA), 80 units/ml gentamicin and 100 units/ml streptomycin. The cells were maintained in an incubator with 5% CO_2_ at 37°C (CO_2_ incubator, MCO-15AC; Sanyo Electric Biomedical Co. Ltd., Japan). Cells from the 5th–7th generations were used in the tests, and 24 h after seeding (detailed density is described in each method), all groups of cells were starved for 12hours in serum-free medium prior to experiments.

The power frequency MF exposure system is composed of 3 main parts: a pair of circular horizontal Helmholtz coil plates (20 cm in height, and 20 cm in radius, each plate consists of 150 turns of copper wire), a signal generator (YM1041, Oscillator, Shanghai No.26 Radio Factory), and an amplifier (NA-4181, 250W Hi-Fi Power Amplifier, FEIYUE). There is a constant-field central area between the plates (10 cm in height, and 6 cm in radius as measured by a Gauss-meter), the field is considered uniform within this cylinder where the samples are placed in the middle platform. The MF employed in this study was 0.4 mT, 50 Hz, the uniformity over which is (0.400±0.012) mT calculated from values measured from different sites of above mentioned region, as described previously [24]. The Helmholtz coils were placed in a CO_2_ culture incubator at 37°C and shielded from external field interactions, and cells and samples were placed in between the plates. The incubator was grounded by an extended wire, which connected the incubator to the ground. The negative control cells (without field exposure) were placed in a separated incubator at the same condition without coils for indicated time length. All samples were divided for following conditions unless indicated elsewhere: (1) sham-exposed; (2) treated with EGF or other factors as indicated (positive control or controls); (3) field-exposed; (4) pre-treated with indicated agents or factors then exposed to the field, (5) without exposure (negative control,N-con). All groups had multiple parallel samples in repeated experiments, and performed blindly by multiple personnel. All groups were carried out at 37°C for 30 min or as indicated. Detailed information of statistics for parallel samples and conditions were listed in Table 1. Protein or cell samples were of 10 µg/ml G-actin on mica for AFM experiments or 5×10^4^/ml or indicated population of cells on glass coverslips for the rest, in conventional 6-well plates (34.8 mM in diameter), were placed in an environment of 5% CO_2_ and 95% air at 22°C or 37°Cfor G-actin and cells, respectively. Each independent experiment was repeated m times resulting in n samples as shown in Table 1, and the number of analyzed cells of each condition was given.

### 3. Detecting F-actin Fiber Assembly in vitro with an Atomic Force Microscope (AFM)

G-actin was dissolved in G-buffer (2 mM Tris-Cl, pH 7.5, 0.2 mM CaCl_2_, 0.5 mM DTT, 0.2 mM ATP) at a concentration of 1 mg/ml and stored at 4°C. The solution was diluted with F-buffer (5 mM Tris-Cl, 2 mM MgCl_2_, 100 mM KCl, 1 mM DTT, 1 mM ATP, 1 nM phalloidin-TRITC, pH 7.5) at 1∶100 and dropped onto newly cleaved mica slits. Immediately following polymerization, which was initiated at room temperature, the slits were randomly divided in several groups as indicated in the section Cell preparation and power frequency MF exposure. The samples were then flushed with 250 mM ammonium acetate at pH 7.2 and dried for AFM [26] (Digital Instruments Corporation, Nanoscope IIIa, Santa Barbara, CA) examination. Tapping scanning mode was used to collect the protein images. This experiment was repeated for 4 times (Table 1).

### 4. Cell Morphology Observations by Scanning Electronic Microscopy (SEM)

FL cells were seeded on coverslips in 6-well cell culture plates at a density of 10^4^/mL and were divided into several groups of 100 nM EGF (positive control), sham-exposed, field-exposed, pre-treated with 1 µM PD for 2 h then exposed to the field, and without exposure (negative control, N-con), or as indicated. Following the aforementioned treatments, the cells were washed in PBS and fixed in 1% OsO_4_ in Millonig’s buffer, dehydrated through a graded acetone series and critical point-dried with CO_2_ in a critical point drier. The specimens were then coated with gold in a sputter instrument and observed using a JXA-840 SEM [25]. Experiments of each group were repeated for 6 times and 34 cells were analyzed except those under PD conditions (3 repeats with 12 cells analyzed) and N-con conditions (3 repeats with 10 cells analyzed), each repeat with 2 parallel samples (Table 1).

### 5. Detecting Focal Adhesion Spots, F-actin Arrangement, Fascin and Arp3 by Confocal Microscopy

FL cells were seeded on coverslips in 6-well cell culture plates at a density of 10^4^/mL and were divided into groups as indicated in the section Cell Preparation and Power Frequency MF Exposure. The cells were then rinsed with PBS, fixed immediately in 4% paraformaldehyde at room temperature for 10 min and gently rinsed with PBS 3 times for 5 minutes each time. The cells were pre-treated with 0.22%Triton-100 for 10 min, gently rinsed with PBS 3 times for 5 min each time, followed by incubating with anti-vinculin, or anti-fascin, or anti-Arp3 first antibody for 1 h at room temperature and then the second antibody-Alexa-488 for 1 h in the dark at 4°C. To mark F-actin, cells were divided as described in the section of Cell Culture and MF Exposure, the rest were pre-treated with 1 µM PD (+PD) or pre-treated with PD then exposed to the MF (+PD+MF). 2 µg/mL phalloidin-TRITC was added to stain F-actin for 1 hour in the dark. Afterward, the cells were gently rinsed 3 times swith PBS, for 10 min each time. Then, the coverslips were sealed. All samples were then observed under the laser scanning confocal microscope [25] (Olympus). The relative FIs in each resultant picture were calculated. The software ImageJ was employed to analyze the fluorescence intensity (FI) of each cell (mean±SD). The method of analysis was described elsewhere. Each independent experiment was repeated as shown in Table 1, and the number of analyzed cells of each condition was given.

### 6. Measuring Total F-actin and [Ca^2+^]_i_ Contents by Flow Cytometry Analysis

FL cells were divided into 3 groups, which were subjected to the5 treatments as indicated in the section of Cell Preparation and Exposure. All groups were exposed or treated as indicated. Then the cells were rinsed with PBS and fixed immediately in 4% paraformaldehyde at 4°C overnight. They were then scraped down and rinsed with PBS 3 times for 10 minutes each time using a tabletop centrifuge and pre-treated with 0.22%Triton-100 for 10 min (to stain Ca^2+^, cells were not pre-treated with Triton-100). Afterwards, the cells were washed again in PBS. The cells were labeled overnight with 2 µg/mL phalloidin-TRITC to stain F-actin or incubated with 24.8 µg/ml Fluo-3/AM [50]–[52] for 40 min at 37°C in the dark to stain Ca^2+^. The samples were then washed with PBS 3 times for 10 minutes each time using a tabletop centrifuge. A 0.5 ml (1×10^5^/ml) cell suspension was collected for each group for flow cytometry measurements (FACScan, Becton Dickson, Franklin Lakes, NJ, USA; EX/EM: 506 nm/525 nm for Fluo-3/AM, and 545 nm/570 nm for phalloidin-TRITC). The mean fluorescence intensities (FI) were normalized by cell number. Each condition was analyzed using Cell Quest software (FACScan, Becton Dickson Company). The mean±SD of FI of each group was calculated, and the relative FIs were compared (Table 1, and Table 2).

### 7. Western Blotting Assay

FL cells were divided into groups as indicated in the above Section of Cell Preparation and Field Exposure. Then, the cells were rinsed with PBS immediately and scraped into LAS2 buffer (G-actin/F-actin In Vivo Assay Kit BK037) at 37°C. If intended for the F/G-actin ratio assay, the sham-exposed group was divided into 3 additional subgroups: (3) sham-exposed; (4) 1 µM cytochalasin-D and (5) 1 µM phalloidin. After gently homogenizing cells for 10 min to lyse the cells and obtaining lysate swith the G-actin/F-actin In Vivo Assay Kit, the lysates were centrifuged at 2,000 rpm for 5 min to pellet the unbroken cells. The supernatants were centrifuged at 100,000g 37°C (Beckman Optima TLX Ultracentrifuge). F-actin was present in the precipitate, while soluble G-actin and most free proteins were found in the supernatants. Each precipitate was dissolved in a volume of 1 µM cytochalasin-D equal to the supernatant on ice [53]. Same volumes of supernatant and precipitate were loaded from the same sample when performing the WB assay, and then were marked with the required primary and secondary antibodies. Then, the gray values from each group were measured by software ImageJ, and normalized by the gray value of the corresponding reference protein (GAPDH). Then the relative gray values were compared with the corresponding sham values. The percent change for each protein was obtained with each sham value as 1. Each experiment was repeated m times resulting in n samples as shown in Table 1.

### 8. Cell Migration

Cell migration experiment, and migration area or rate calculations were carried out according the method of a reported paper [54]. FL cells of 5–7 generation were seeded in culture dishes (r = 3 cm) at a density of 10^4^/mL. Then the cells were starved in serum-free medium prior to experiments when they covered 90% of each dish. 8 hours later, one scratch (blank) crossing the dish centre with a marker nearby was made on each dish with 100 µL pipette tips [54], washed by 2 mL PBS 3 times, then incubated in serum-free medium for another 4 hours (cells were pre-starved for 12 h in all). The dishes were randomly divided into two large sets, sham or MF exposed to a 0.4 mT MF or under the conditions as indicated. Photographs were taken at the same vision field according to the markers by 40× microscopy (XDS200 inverted microscope, Phenix) before (0 hour) exposure treatment, and when MF or sham exposed for 3, 6, 12, 18, or 24 hours, respectively under the indicated conditions (Fig. 8). The cell free surface areas of the initial state (blank 0) were measured by software ImageJ, and the migrated areas (×10^3^ µm^2^, mean ±SD) by migrating cells were calculated by the formula of surface area blank 0 minus surface area of cell free (blank t) at 3, 6, 12, 18, or 24 hours. Migration rates (×10^3^ µm^2^/h, mean ±SD) were the average migrated surface areas by cells relative to the time (h). Detailed information of repeating and parallel samples was given in Table 1.

### 9. Transfection and Shedding Assay

FL cells were transfected with p-APtag-TGF-α at 90–95% confluency using LipofectamineTM 2000(Invitrogen). 24 hours later, the cells were washed with PBS, and then, 1 ml fresh Opti-Mem was added. After 0.5 hours, the supernatant was collected, and fresh Opti-Mem with or without 25 ng/ml PMA was added. Then, the cells were exposed to a 0.4 mT 50 Hz magnetic field for 0.5 hours. In the sham group, fresh Opti-Mem with or without 25 ng/ml PMA was added, and the cells were incubated under conditions identical to the first half hour. The supernatant was collected at the end of 0.5 hour exposure time. An alkaline phosphatase detection system was used to monitor shedding of TGF-α, as described previously [55]–[56]. In brief, 100 µl of cleared supernatant from either unstimulated or stimulated cells was added to separate wells in a 96-well microplate. Then, 100 µl of 2 mg/mL 4-nitrophenylphosphate (4-NPP) in water was added to each well. 4-NPP is a substrate of alkaline phosphatase, which is converted into nitrophenol (yellow color), resulting in increased absorbance at 405 nm. The supernatant–substrate mix was incubated at 37°C for color development (yellow). Supernatant from non-transfected cells incubated with4-NPP for the same amount of time was used as the spectrophotometric blank. The absorbance at 405 nm correlated with the amount of shed TGF-α in the cell supernatant. The experiments were repeated 3 times. Detailed information of repeating and parallel samples was given in Table 1.

### 10. Software ImageJ Analysis of Cytoskeleton Gray Values and Statistics

Software ImageJ 1.46 is an open and free software developed by National Institutes of Health (NIH, http://rsb.info.nih.gov/ij/download.html), in Bethesda, Maryland, USA. In this paper, software ImageJ was used to calculate the information of the area and the pixel value statistics of the defined selections. Using this software, the fluorescent intensities corresponding to the immuno-fluorescence photographs and the gray values of the bands corresponding to protein contents were also quantified by extracting the gray values for indicated FIs in each cell, or the cell areas, or the gel bands, respectively.

For the data from FIs of immuno-fluorescence photographs and flow cytometry, each normalized results were calculated from the formula of *changed percent = ((the test gray value-the corresponding Sham gray value)/the Sham gray value)*, and the percent changes in fluorescent intensity for each protein were obtained.

The results were presented as mean±SD of number of repeated times or samples or analyzed cells for data analysis (Table 1), while the WB results were shown by percentages of changes. Students’t-test was employed to perform statistical comparisons between groups and the P values were obtained. Differences were considered statistically significant at P<0.05.
